# Surgical treatment of primary cardiac tumors in the contemporary era: A single‐centre analysis

**DOI:** 10.1111/jocs.15813

**Published:** 2021-07-12

**Authors:** Matteo Matteucci, Sandro Ferrarese, Vittorio Mantovani, Daniele Ronco, Federica Torchio, Cinzia Franzosi, Jacopo Marazzato, Roberto De Ponti, Roberto Lorusso, Cesare Beghi

**Affiliations:** ^1^ Department of Surgical and Morphological Sciences, Circolo Hospital University of Insubria Varese Italy; ^2^ Department of Cardiothoracic Surgery, Heart and Vascular Centre Maastricht University Medical Centre Maastricht The Netherlands; ^3^ Department of Medicine and Surgery, Circolo Hospital University of Insubria Varese Italy

**Keywords:** cardiac tumors, myxoma, surgical resection

## Abstract

**Background:**

Primary cardiac tumors (PCT) are rare lesions but have the potential to cause significant morbidity if not timely treated. We reviewed our single‐center experience in the surgical treatment of PCT with a focus on the long‐term outcome.

**Methods:**

From 2001 to 2020, 57 consecutive patients underwent surgical resection of PCT at our Institution. Data including the demographic characteristics, tumor histology, surgical procedure, and postoperative outcomes were collected and analyzed.

**Results:**

Mean age at presentation was 63.6 ± 11.2 years, and 33 (57.9%) of the patients were female. A total of 55 (96.5%) subjects were diagnosed with benign cardiac tumor, while the remaining had malignant tumors. The most common histopathological type was myxoma. All patients survived to hospital discharge. Main postoperative complications were: acute kidney injury (*n *= 3), sepsis (*n *= 3), and stroke (*n* = 2). Mean follow‐up time was 9 ± 5.9 years. Long‐term mortality was 22.8% (13/57). No tumor recurrence was observed among survivors. There was a significant relationship between mortality and pathological characteristics of the tumor, and myxomas had higher survival rates.

**Conclusion:**

Surgical treatment of PCT is a safe and highly effective strategy associated with excellent short‐term outcomes. Long‐term survival remains poor for primary malignant tumors of the heart.

## 1 INTRODUCTION

Primary cardiac tumors (PCT) are a rare disease, with reports of prevalence from autopsy series ranging from 0.001% to 0.3%.^1^, ^2^ However, they constitute a major diagnostic and therapeutic challenge. Recently, considerable advances in cardiac imaging have made the diagnosis of heart neoplasms accurate and readily available. Hence, the clinical interest on PCT has been progressively increasing. Usually, over 75% of these tumors are benign, with atrial myxomas being the most common.^3^ Cardiac sarcoma represents the most frequent primary malignant tumor of the heart.^4^ Surgical resection is the most common and effective therapy for both benign and malignant PCT. Because of the rarity of PCT occurrence, only few studies have been published describing the presentation and outcomes after surgical management. Moreover, the majority of these reports focus on patients submitted to tumor resection in the past half century. The aim of the present study was to review our experience in the surgical treatment of PCT over the last two decades, with a focus on the long‐term outcomes.

## 2 MATERIALS AND METHODS

We conducted an observational retrospective study of adult (>18 years old) patients with pathological confirmation of PCT undergoing surgical resection at our institution between January 1, 2000 and December 31, 2020. Subjects with metastatic tumors of the heart, and those in whom a primary cardiac origin was not clear, were excluded from the analysis. Preoperative diagnosis was established using transthoracic echocardiogram (TTE) or other cardiac imaging techniques such as computed tomography (CT) or magnetic resonance (MRI). Information including demographics, comorbidities, symptoms, tumor location, pathological findings, operation information, and in‐hospital outcomes were collected from the hospital database. Data about postdischarge death was obtained from the Regional Institutional Health Database System. All survivors were contacted via telephone to assess late functional status and tumor recurrence. The study was conducted in accordance with the principles of the declaration of Helsinki and the study protocol was approved by the institution ethical committee (nr. 164/2020).

### 2.1 Statistical analysis

Categorical data were described as numbers and percentages, while continuous variables were presented as mean ± *SD* or median and interquartile range. Survival was estimated using the Kaplan–Meier method and curves were compared using the log‐rank test. Statistical analysis was performed with SPSS for Windows, version 23 (IBM Corporation). A value of *p* < .05 was considered to be statistically significant.

## 3 RESULTS

### 3.1 Patient demographics and tumor characteristics

**Figure 1:**
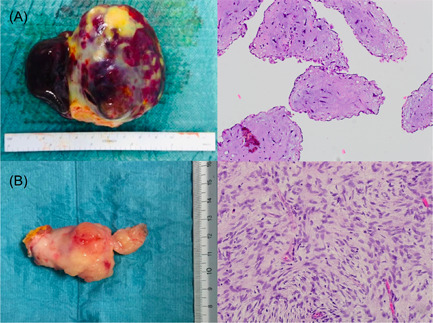
Macroscopic appearance and histology of primary cardiac tumors. (A) Surgical specimen (measured 3.5 × 7 cm) and histology (polygonal cells with eosinophilic cytoplasm scattered throughout myxoid matrix) of an atrial myxoma. (B) Surgical specimen (measured 3.2 × 5 cm) and histology (tumor cells with marked nuclear polymorphism and high mitotic activity) of an intimal sarcoma (all samples were stained with hematoxylin‐eosin; original magnification: ×20)

A total of 57 patients underwent surgical rection for PCT during the study period. Baseline characteristics are shown in Table 1. Overall, patient mean age at tumor diagnosis was 63.6 ± 11.2 years, and 57.9% of the subjects were women. Cardiovascular risk factors included hypertension (56.1%), dyslipidaemia (29.8%), diabetes mellitus (17.5%), and smoking (14%). The most common presenting complaints were dyspnea (26.3%), cerebral ischemia (19.3%), palpitations (17.5%), and chest pain (12.8%). Approximately one‐third of the individuals were asymptomatic. The cardiac function of most patients was normal; only 19.3% of subjects presented in NYHA class III or IV. The majority of patients were diagnosed with echocardiography (93%), with cardiac MRI (8.8%) and CT (12.3%) used in the minority of the patients. Benign tumors accounted for 96.5% of diagnosed PCT, with myxoma being the most common (75.4%) (Figure 1A), followed by papillary fibroelastoma (19.3%). Among the primary malignancies, sarcomas were the only ones observed (Figure 1B). There was a substantial variation in tumor histology by tumor location; left and right atrial tumors were primarily myxomas, whereas valvular tumors were predominantly papillary fibroelastoma. The mean maximum diameter of lesions was 3.4 ± 1.9 cm according to echocardiogram measurements. Tumor characteristics are shown in Table 2.

**Table 1:** Patients’ baseline characteristics

Characteristics	Value
Demographic data	
Patients	57
Age (years)	63.6 ± 11.2
Male	24 (42.1%)
BMI (kg/m^2^)	26.2 ± 4.2
LVEF (%)	55.7 ± 4.3
NYHA Class 3/4	11 (19.3%)
Comorbidities	
Hypertension	32 (56.1%)
Dyslipidaemia	17 (29.8%)
Diabetes mellitus	10 (17.5%)
Smoking	8 (14%)
COPD	5 (8.8%)
Thyroid disease	11 (19.3%)
Atrial fibrillation	7 (12.3%)
Coronary artery disease	10 (17.5%)
Symptoms	
Dyspnea	15 (26.3%)
Chest pain	7 (12.3%)
Palpitations	10 (17.5%)
Cerebral ischemia	11 (19.3%)
Fever	5 (8.8%)

*Note*: Data are shown as mean ± *SD* or number (%) as appropriate.

Abbreviations: BMI, body max index; COPD, chronic obstructive pulmonary disease; LVEF, left ventricular ejection fraction; NYHA, New York Heart Association.

**Table 2:** Tumor characteristics categorized by histology

	Myxoma (*n* = 43)	Fibroelastoma (*n* = 11)	Lipoma (*n* = 1)	Sarcoma (*n* = 2)
Dimension (cm)^a^	3.8 ± 1.9	1.4 ± 0.5	5.8	4.7 ± 1.8
Location				
Left atrium	36 (83.7%)	1 (9.1%)	0	1 (50%)
Right atrium	2 (4.7%)	1 (9.1%)	0	0
Left ventricle	0	0	0	0
Right ventricle	1 (2.3%)	0	0	0
Aortic valve	1 (2.3%)	3 (27.3%)	0	0
Mitral valve	3 (7%)	5 (45.4%)	0	0
Tricuspid valve	0	1 (9.1%)	0	0
Great arteries	0	0	1 (100%)	1 (50%)

^a^ Maximum diameter detected at preoperative imaging assessment.

### 3.2 Surgical approach and operative data

Surgical tumor resections was performed through standard median sternotomy in all cases. The site of cannulation depended mainly on the location of the lesion. Operative information is presented in Table 3. Bicaval cannulation was used in 51 (89.5%) patients, followed by two‐stage cannulation in 5 cases, and femoral cannulation in 1 subject. The majority of atrial, mitral valve, and tricuspid valve tumors were resected by a single atrial approach, whereas aortic valve tumors were commonly approached via aortotomy. Twenty‐five subjects (43.9%) required cardiac reconstruction with prosthetic material (pericardial or Dacron) after tumor removal. The 2 patients with sarcoma underwent complete resection in a standard fashion; cardiac autotransplantation was not performed. One patient with tumor originating in the main pulmonary artery (PA) required reconstruction of PA with prosthetic patch after resection with tumour‐free margins; the other one with sarcoma originating in the left atrium (LA) underwent wide tumor excision and LA reconstruction was accomplished with xenopericardial patch. A total of 17 (29.8%) individuals underwent concomitant cardiac surgical procedure in addition to tumor resection. The most frequently associated procedures performed were coronary artery bypass grafting (15.8%) and mitral valve repair (8.8%). In only 4 cases the concomitant procedure was related to the tumor excision (3 mitral valve repair and 1 tricuspid valve repair due to tumor location involving heart valves). The mean cardiopulmonary bypass time was 82.3 ± 41.3 min and the mean cross‐clamp time was 59.7 ± 30.4 min.

**Table 3:** Intraoperative data

Characteristics	Value
Tumor diameter (cm)	3.8 ± 1.8
Cannulation strategy	
Bicaval	51 (89.5%)
Two‐stage	5 (8.8%)
Femoral vessel	1 (1.7%)
Technique	
Simple tumor resection	32 (56.1%)
Patch reconstruction	25 (43.9%)
Concomitant procedures	
CABG	9 (15.8%)
Aortic valve replacement	4 (7%)
Mitral valve repair	5 (8.8%)
Tricuspid valve repair	2 (3.5%)
Duration	
CPB time (min)	82.3 ± 41.3
Aortic cross‐clamp time (min)	59.7 ± 30.4

*Note*: Data are shown as mean ± *SD* (%) as appropriate.

Abbreviations: CABG, coronary artery bypass grafting; CPB, cardiopulmonary bypass.

### 3.3 Postoperative outcomes and survival analysis

**Figure 2:**
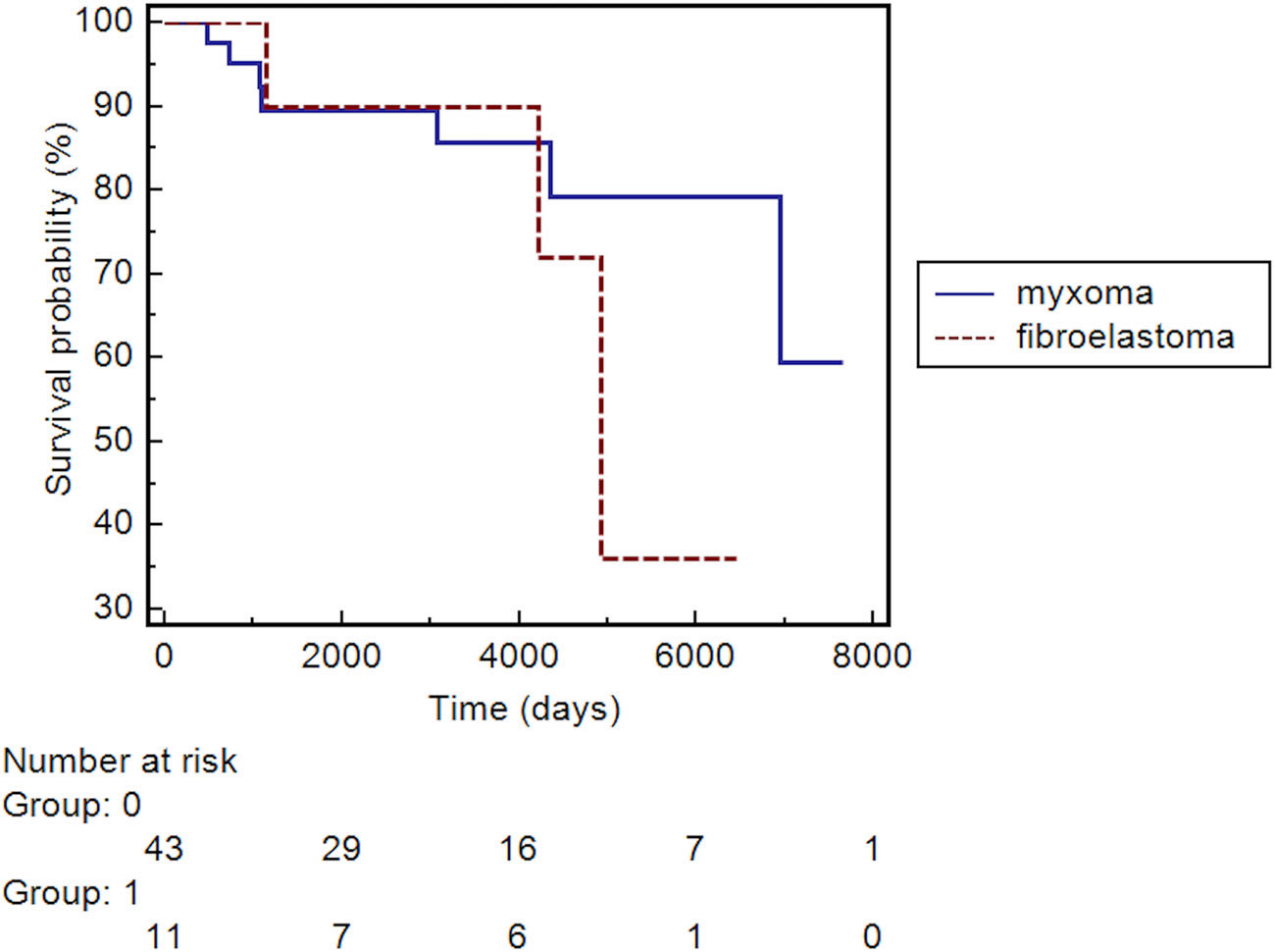
Kaplan–Maier's survival analysis: myxomas versus fibroelastomas. Analysis showed that after a relative equal survival between myxomas and fibroelastomas in the first year of follow‐up, patients with papillary fibroelastoma had poor long‐term survival when compared to subjects who underwent myxoma excision; however, this trend did not reach statistical significance (*p* = .41)

Postoperative outcomes are reported in Table 4. The mean intensive care unit length‐of‐stay was 2.3 ± 2.9 days. The main in‐hospital postoperative complications were: AKI (*n* = 3), sepsis (*n* = 3), and stroke (*n* = 2). Eleven patients (19.3%) required inotropes due to low cardiac output after surgery; no subjects necessitated intra‐aortic balloon pump or extracorporeal membrane oxygenation support postoperatively. All patients survived to hospital discharge. The mean length‐of‐hospitalization was 8.3 ± 5.2 days. Follow‐up was obtained in 100% of patients at a mean of 9 ± 5.9 years. Overall, long‐term survival was 77.2%. The individuals diagnosed with malignant tumors were all dead at the end of the follow‐up period (maximum survival: 22 months). One patient died of distant metastases within 2 years, whereas the other died 17 months postoperatively of intracerebral hemorrhage without disease progression. Main characteristics of patients with malignant tumors are illustrated in Table 5. No tumor recurrence requiring reoperation was observed among survivors. At the time of follow‐up, only 4.5% (2/44) of patients presented a NYHA Class III or IV. Cumulative survival for patients diagnosed with benign tumors was compared based on tumor histological subtypes (myxoma vs. fibroelastoma), and is presented in Figure 2. Analysis revealed that patients with papillary fibroelastoma had poorer long‐term survival when compared to subjects who underwent myxoma resection, however this trend did not reach statistical significance (*p* = .41).

**Table 4:** Postoperative results

Characteristics	Value
Early outcomes	
ICU length of stay (days)	2.3 ± 2.9
Hospital length of stay (days)	8.3 ± 5.2
In‐hospital death	0
Postoperative complications	
Atrial fibrillation	29 (50.9%)
Stroke	2 (3.5%)
AKI	3 (5.4%)
Urinary tract infection	6 (10.5%)
Pneumonia	2 (3.5%)
Sepsis	3 (5.4%)
Inotropes	11 (19.3%)
Permanent pacemaker	3 (5.4%)
Late outcomes	
Follow‐up time (year)	9 ± 5.9
Death after discharge	13 (22.8%)
Cardiac tumor recurrence^a^	
NYHA Class 3/4^a^	

*Note*: Data are shown as mean ± *SD* or number (%) as appropriate.

Abbreviations: AKI, acute kidney injury; ICU, intensive care unit; NYHA, New York Heart Association.

^a^ For survivors only.

**Table 5:** Clinical findings, surgical procedures, and follow‐up of patients with cardiac sarcoma

	Age (year)/gender	Symptoms	Size (cm)	Location	Approach	Surgery	Follow‐up (cause of death)
1	68/F	Dyspnea AF	3.2 × 5	LA	Left atriotomy	Complete resection + patch repair	Died, 23 months IC hemorrhage
2	70/F	Dyspnea	3.5 × 6	mPA	Pulmonary arteriotomy	Complete resection + patch repair	Died, 17 months (metastases)

Abbreviations: AF, atrial fibrillation; cm, centimeters; F, female; IC, intracerebral; LA, left atrium; M, male; mPA, main pulmonary artery.

## 4 DISCUSSION

Despite the increase availability of several cardiac imaging modalities, cardiac tumors remain an exceptionally rare entities, representing the cause of only 0.3%–0.85% of all open‐heart surgeries.^1^, ^5^ Our study corroborates previous observations suggesting that PCTs occur more often in the middle‐aged population, and the morbidity is higher in female.^6^ Atrial myxomas are confirmed to be the most common PCT. Although significantly less frequent, papillary fibroelastoma is the second‐most common primary tumor of the heart. As previously described,^6^, ^7^ malignant PCT are rarely encountered, accounting for less than 4% of cases in our series. Individuals with PCT have diverse clinical presentation. The majority of patients usually present with dyspnea or embolic symptoms, such as transient ischemic attack or stroke.^6^, ^8^ According to our experience, 35% of subjects were asymptomatic, and shortness of breath was the most common symptom, followed by cerebrovascular accidents (CVAs). One interesting observation was the high prevalence of thyroid disease (20%) in the patient cohort; this association should be examined in further analysis for establishing potential screening recommendations.

Improvements in cardiac imaging methods over the last two decades have facilitated the diagnosis of cardiac tumors. Transthoracic echocardiography (TTE) is now the principal imaging technique for cardiac mass detection.^9^ Transesophageal echocardiography (TEE) may be required for more comprehensive assessment of the relationship of mass with surrounding structures and valve competence.^9^ CT and RMI are generally reserved for patients in whom echocardiography is insufficient for diagnosis; moreover, they are particularly helpful to assess tumor extension and to facilitate preoperative planning.^10^ Accordingly, TTE was our main diagnostic tool, aiding in determining tumor size and location. A small number of subjects received TC, MRI or TEE. The decision to use advanced imaging methods was based on the suspected tumor type and clinical manifestation.

In patients with benign cardiac neoplasms or in those with resectable primary malignant disease and no evidence of metastasis, surgery is generally indicated to improve prognosis.^11^ For selected subjects, other approaches such as heart transplantation^12^ or heart autotransplantation^13^ should be considered. The surgical approach depends on the location and size of the tumor. Some authors have suggested both right and left atriotomy to allow inspection of all four chambers during tumor resection.^14^ We believe that visual examination of all cardiac chambers is unnecessary if a detailed examination of the TTE, or other more advanced imaging, is performed preoperatively. Indeed, in our series most patients underwent right or left atriotomies. Biatrial incision was accomplished only when evaluation of the relationship between the mass and the cardiac chambers on TEE was not clear, or if adequate exposure for complete resection was not achieved through a single atrial approach. Another controversial issue concerns the extent of resection. Although authors have suggested extensive excision and patch repair,^15^ particularly in the suspicion for malignancy, others have argued that a simple excision is usually safe and effective.^16^ Elbardissi et al.^6^ have provided evidence that tumor recurrence may be attributable to a patient's biological propensity rather than the method of surgical excision. Our policy is to excise the tumor completely with an acceptable safety margin. In this study, patch repair was performed in almost 45% of cases due to the need for wide excision. Based on our own experience, we observed no recurrences during follow‐up in survivors.

After surgical resection, the main difference between benign and malignant tumors is the survival rates.^6^, ^17^, ^18^ In our series, surgical resection of PCT has good short and long‐term results; conversely, the prognosis of malignant types is poor. The median survival of patients with malignant tumors was less than 2 years. These findings are consistent with a previous report that documented survival of 16.5 months.^19^

Primary cardiac sarcomas possess the highly aggressive local growth and metastatic spreads are relative common. The prognosis of patients is primarily related with tumor histology, complete or incomplete resection and distant organ metastasis at initial diagnosis.^20^ Clinical presentation of heart sarcoma may vary depending on location. Studies have found that right‐sided cardiac sarcomas tend to be bulky, are more infiltrative and metastasize earlier;^21^ thus, clinical symptoms are commonly nonspecific. In contrast, left‐sided cardiac sarcomas are less invasive, more circumscribed, and usually cause heart failure early in the disease process.^21^ Sarcomas that originate in the pulmonary arteries are the rarest group, and generally present with symptoms related to intraluminal obstruction such as dyspnea. Our approach to malignant PCT is conservative and consists of surgical removal without extensive cardiac resection. While some have advocated for highly aggressive procedures including cardiomyoplasty and autotransplantation for the resection of complex heart tumors,^13^, ^22^ it is currently unknown whether these approaches result in superior survival outcomes. As such, malignant PCT present the greatest challenge to cardiac surgeons.

In accord with previous studies,^6^, ^8^ we found that individuals with papillary fibroelastoma had poorer long‐term results compared with subjects diagnosed with myxoma, albeit not statistically significance. This could be explained through the fact that the patients with fibroelastoma were older (64 vs. 62 years old) and had more CVAs at the time of surgical intervention (27% vs. 11%). The increased occurrence of CVA in subjects with fibroelastoma is not a novel findings; we have found previous observations in studies by Tamin et al.^23^ and Gowda et al.^24^ Indeed, the rate of concomitant surgical procedures was greater in patients underwent fibroelastoma excision (63% vs. 18%). Thus, it was a sicker group than myxoma group and, not surprisingly, had a different postoperative long‐term survival.

Overall, the patient population presented in this study showed acceptable postoperative course with results comparable to the other nontumor cardiac surgical patients, However, we observed a slightly higher incidence of postoperative complications (e.g., stroke, AKI, atrial fibrillation), when compared with previous publications on this topic.^8^, ^25^ This may be because of the higher rate of concomitant procedures performed in addition to tumor resection in our experience. Noticeably, performing concomitant procedures during tumor have a negative effect on postoperative morbidity.

### 4.1 Limitations

The major limitations of the current study were the retrospective design and its small sample size because of the rarity of PCT. Moreover, we only evaluated patients underwent surgical resection; conservatively managed heart tumors were not considered. Because of the unavailability of data on cause of death, only all‐cause mortality was assessed. We had no tumor recurrences at follow‐up, however, recurrence was assessed only in survivors due to failed in obtaining deceased patients’ medical records. Last, since the current study was not designed to detect confounders that impact survival, it is important to note that our results only described the association with long‐term survival rather than causation.

## 5 CONCLUSION

PCT are challenging because they are extremely are. The first‐choice treatment is surgical excision, which must be performed as early as possible to reduce the risk of embolic events. Surgical resection of benign tumors is safe and, especially for myxomas, provides excellent long‐term prognosis. On the contrary, patients with malignant tumors have a dismal prognosis.

## CONFLICT OF INTERESTS

Roberto Lorusso consultant for Medtronic and LivaNova, and member of the Advisory Board of Eurosets and PulseCath. All other authors have no conflict of interests.

## References

[1] Patel J , Sheppard MN . Pathological study of primary cardiac and pericardial tumours in a specialist UK Centre: surgical and autopsy series. Cardiovasc Pathol. 2010;19(6):343‐352. 19747857 10.1016/j.carpath.2009.07.005

[2] Lam KY , Dickens P , Chan AC . Tumors of the heart. A 20‐year experience with a review of 12,485 consecutive autopsies. Arch Pathol Lab Med. 1993;117(10):1027‐1031. 8215825

[3] McAllister HA. Jr. , Hall RJ , Cooley DA Tumors of the heart and pericardium. Curr Probl Cardiol. 1999;24(2):57‐116. 10028128

[4] Shapiro LM . Cardiac tumours: diagnosis and management. Heart. 2001;85(2):218‐222. 11156679 10.1136/heart.85.2.218 PMC1729629

[5] Strecker T , Rösch J , Weyand M , Agaimy A . Primary and metastatic cardiac tumors: imaging characteristics, surgical treatment, and histopathological spectrum: a 10‐year‐experience at a German heart center. Cardiovasc Pathol. 2012;21(5):436‐443. 22300501 10.1016/j.carpath.2011.12.004

[6] Elbardissi AW , Dearani JA , Daly RC , et al. Survival after resection of primary cardiac tumors: a 48‐year experience. Circulation. 2008; 118(14 Suppl):S7‐S15. 18824772 10.1161/CIRCULATIONAHA.107.783126

[7] Li S , Gao Ch . Surgical experience of primary cardiac tumor: single‐institution 23‐year report. Med Sci Monit. 2017;23:2111‐2117. 28469127 10.12659/MSM.903324 PMC5426384

[8] Jawad K , Owais T , Feder S , et al. Two decades of contemporary surgery of primary cardiac tumors. Surg J (N Y). 2018;4(4):e176‐e181. 30345368 10.1055/s-0038-1673333 PMC6191300

[9] Butany J , Nair V , Naseemuddin A , Nair GM , Catton C , Yau T . Cardiac tumours: diagnosis and management. Lancet Oncol. 2005;6(4):219‐228. 15811617 10.1016/S1470-2045(05)70093-0

[10] Liddy S , McQuade C , Walsh KP , Loo B , Buckley O . The assessment of cardiac masses by cardiac CT and CMR including pre‐op 3D Reconstruction and Planning. Curr Cardiol Rep. 2019;21(9):103. 31367849 10.1007/s11886-019-1196-7

[11] Bruce CJ . Cardiac tumours: diagnosis and management. Heart. 2011;97(2):151‐160. 21163893 10.1136/hrt.2009.186320

[12] Li H , Yang S , Chen H , et al. Survival after heart transplantation for non‐metastatic primary cardiac sarcoma. J Cardiothorac Surg. 2016;11(1):145. 27716444 10.1186/s13019-016-0540-x PMC5048623

[13] Blackmon SH , Patel AR , Bruckner BA , et al. Cardiac autotransplantation for malignant or complex primary left‐heart tumors. Tex Heart Inst J. 2008;35(3):296‐300. 18941651 PMC2565530

[14] Jones DR , Warden HE , Murray GF , et al. Biatrial approach to cardiac myxomas: a 30‐year clinical experience. Ann Thorac Surg. 1995;59(4):851‐855. 7695408 10.1016/0003-4975(95)00064-r

[15] Gerbode F , Kerth WJ , Hill JD . Surgical management of tumors of the heart. Surgery. 1967;61(1):94‐101. 6016669

[16] Melo J , Ahmad A , Chapman R , Wood J , Starr A . Primary tumors of the heart: a rewarding challenge. Am Surg. 1979;45(11):681‐683. 517866

[17] Kośmider A , Jaszewski R , Marcinkiewicz A , Bartczak K , Knopik J , Ostrowski S . 23‐year experience on diagnosis and surgical treatment of benign and malignant cardiac tumors. Arch Med Sci. 2013;9(5):826‐830. 24273564 10.5114/aoms.2013.38677 PMC3832829

[18] Kuplay H , Kurç E , Mete EM , et al. Early and late results in surgical excision of primary cardiac tumors: our single‐institution experience. Turk Gogus Kalp Damar Cerrahisi Derg. 2018;26(2):177‐182. 32082732 10.5606/tgkdc.dergisi.2018.14985 PMC7024111

[19] Basso C , Valente M , Poletti A , Casarotto D , Thiene G . Surgical pathology of primary cardiac and pericardial tumors. Eur J Cardiothorac Surg. 1997;12(5):730‐737. 9458144 10.1016/s1010-7940(97)00246-7

[20] Poterucha TJ , Kochav J , O'Connor DS , Rosner GF . Cardiac tumors: clinical presentation, diagnosis, and management. Curr Treat Options Oncol. 2019;20(8):66. 31250250 10.1007/s11864-019-0662-1

[21] Ramlawi B , Leja MJ , Abu Saleh WK , et al. Surgical treatment of primary cardiac sarcomas: review of a single‐institution experience. Ann Thorac Surg. 2016;101(2):698‐702. 26476808 10.1016/j.athoracsur.2015.07.087

[22] Chachques JC , Argyriadis PG , Latremouille C , et al. Cardiomyoplasty: ventricular reconstruction after tumor resection. J Thorac Cardiovasc Surg. 2002;123(5):889‐894. 12019373 10.1067/mtc.2002.121493

[23] Tamin SS , Maleszewski JJ , Scott CG , et al. Prognostic and bioepidemiologic implications of papillary fibroelastomas. J Am Coll Cardiol. 2015;65(22):2420‐2429. 26046736 10.1016/j.jacc.2015.03.569

[24] Gowda RM , Khan IA , Nair CK , Mehta NJ , Vasavada BC , Sacchi TJ . Cardiac papillary fibroelastoma: a comprehensive analysis of 725 cases. Am Heart J. 2003;146(3):404‐410. 12947356 10.1016/S0002-8703(03)00249-7

[25] Mkalaluh S , Szczechowicz M , Torabi S , et al. Surgical treatment of cardiac tumors: insights from an 18‐year single‐center analysis. Med Sci Monit. 2017;23:6201‐6209. 29289957 10.12659/MSM.905451 PMC5757895

